# Modeling and Reconstruction of Mixed Functional and Molecular Patterns

**DOI:** 10.1155/IJBI/2006/29707

**Published:** 2006-01-17

**Authors:** Yue Wang, Jianhua Xuan, Rujirutana Srikanchana, Peter L. Choyke

**Affiliations:** ^1^ Department of Electrical and Computer Engineering, Computational Bioinformatics and Bioimaging Laboratory, Virginia Polytechnic Institute and State University, 4300 Wilson Boulevard, Suite 750, Arlington, VA 22203, USA; ^2^ Department of Electrical Engineering and Computer Science, The Catholic University of America, Washington, DC 20064, USA; ^3^ Riverain Medical, Riverain Research Group, Rockville, MD 20850, USA; ^4^ Molecular Imaging Program, National Cancer Institute, National Institutes of Health, Bethesda, MD 20892, USA

## Abstract

Functional medical imaging promises powerful tools for the visualization and elucidation of important disease-causing biological processes in living tissue. Recent research aims to dissect the distribution or expression of multiple biomarkers associated with disease progression or response, where the signals often represent a composite of more than one distinct source independent of spatial resolution. Formulating the task as a blind source separation or composite signal factorization problem, we report here a statistically principled method for modeling and reconstruction of mixed functional or molecular patterns. The computational algorithm is based on a latent variable model whose parameters are estimated using clustered component analysis. We demonstrate the principle and performance of the approaches on the breast cancer data sets acquired by dynamic contrast-enhanced magnetic resonance imaging.


Hindawi Publishing Corporation
International Journal of Biomedical Imaging
Volume 2006, Article ID 29707, Pages 1–9
DOI 10.1155/IJBI/2006/29707

### Modeling and Reconstruction of Mixed Functional and Molecular Patterns

**Yue Wang,[1] Jianhua Xuan,[2] Rujirutana Srikanchana,[3] and Peter L. Choyke[4]**

[1] *Department of Electrical and Computer Engineering, Computational Bioinformatics and Bioimaging Laboratory, Virginia Polytechnic Institute and State University, 4300 Wilson Boulevard, Suite 750, Arlington, VA 22203, USA*
[2] *Department of Electrical Engineering and Computer Science, The Catholic University of America, Washington, DC 20064, USA*
[3] *Riverain Medical, Riverain Research Group, Rockville, MD 20850, USA*
[4] *Molecular Imaging Program, National Cancer Institute, National Institutes of Health, Bethesda, MD 20892, USA*

Received 10 August 2005; Accepted 23 October 2005

Recommended for Publication by Ming Jiang

Functional medical imaging promises powerful tools for the visualization and elucidation of important disease-causing biological processes in living tissue. Recent research aims to dissect the distribution or expression of multiple biomarkers associated with disease progression or response, where the signals often represent a composite of more than one distinct source independent of spatial resolution. Formulating the task as a blind source separation or composite signal factorization problem, we report here a statistically principled method for modeling and reconstruction of mixed functional or molecular patterns. The computational algorithm is based on a latent variable model whose parameters are estimated using clustered component analysis. We demonstrate the principle and performance of the approaches on the breast cancer data sets acquired by dynamic contrast-enhanced magnetic resonance imaging.

Copyright © 2006 Yue Wang et al. This is an open access article distributed under the Creative Commons Attribution License, which permits unrestricted use, distribution, and reproduction in any medium, provided the original work is properly cited.

### 1. INTRODUCTION

Functional imagingtechnologies are providing researchers and physicians with exciting new tools to study important disease-causing biological processes in living tissue [1, 2]. Dynamic contrast-enhanced magnetic resonance imaging (DCE-MRI) uses various molecular weight contrast agents to assess tumor vascular permeability and quantify cellular and molecular abnormalities in blood vessel walls [3]. DCE-MRI can characterize vascular heterogeneity and elucidate features that distinguish angiogenic blood vessels from their normal counterparts, and has potential utility in assessing the efficacy of angiogenesis inhibitors in cancer treatment [2–4]. Although DCE-MRI can provide a meaningful estimation of vascular permeability when a tumor is homogeneous, many malignant tumors show markedly heterogeneous areas of permeability and vascular endothelial growth factor (VEGF) expression; thus the signal of each pixel often reflects multiple microenvironments in a tumor representing a complex summation of vascular permeability with various diffusion rates [2, 3].

Several quantitative methods based on parametric compartment modeling (CM) have been developed to dissect the spatial distribution of vascular heterogeneity associated with tumor angiogenesis [3, 5, 6]. These methods estimate the fundamental kinetics components (called factors) and the associated factor weights (called factor images) [6–8]. Each factor is interpreted as the time course of a compartment, whereas each factor image is interpreted as local weights representing the spatial distribution of vascular permeability with different diffusion rates [2, 9]. The parametric model chosen may not fit the data obtained, and each model makes a number of assumptions that may not be valid for every tissue or tumor type. The causes for modeling failures are complex and often not well understood [6, 10]. Key reasons include multiple tissue compartments, an incorrect arterial input function, and numerical nonidentifiability of the parametric model [3, 6, 9–11]. This motivates the consideration of clustered component analysis (CCA) that can be based on a flexible compartment latent variable model [12, 13]. The objective is to factorize the underlying angiogenic permeability distributions (APD) and time activity curves (TAC) from dynamically mixed DCE-MRI image sequences [14, 15].

### 2. THEORY AND METHODS

We first introduce a simple form of compartment latent variable model for DCE-MRI. Without loss of generality, we initially focus on the two-tissue compartment model shown in Figure 1. The tracer characterization within a region of interest can be approximated by a set of first-order differential equations [15]:

$$\begin{aligned} \dot{c}_f(t) &= k_{1f}c_p(t) - k_{2f}c_f(t), \\ \dot{c}_s(t) &= k_{1s}c_p(t) - k_{2s}c_s(t), \\ c(t) &= c_f(t) + c_s(t) + c_p(t), \end{aligned} \quad (1)$$

where $c_f(t)$ and $c_s(t)$ are the tissue activity in the fast turnover and slow turnover pools, respectively, at time $t$; $c_p(t)$ is the tracer concentration in plasma (the input function); $c(t)$ is the measured total tissue activity; $k_{1f}$ and $k_{1s}$ are the unidirectional transport constants from plasma to tissue (permeability in ml/min/g: spatially varying); $k_{2f}$ and $k_{2s}$ are the rate constants for efflux (diffusion in /min: spatially invariant) in the fast flow and slow flow pools, respectively [5–7].

Mathematical consideration based on a latent variable model suggests a simple method to convert temporal kinetics (1) to spatial information [7]. Let $\mathbf{x}(i) = [x(i,t_1), x(i,t_2), \ldots, x(i,t_L)]^T$ be the observed tracer activities of pixel $i$ measured at $L$ time points. Now consider a source vector of spatial permeability distributions $\mathbf{k}(i) = [k_f(i), k_s(i), k_p(i)]^T$ together with an $L\times 3$ *mixing matrix* $\mathbf{A}(t)$ which maps the latent space into the data space: $\mathbf{x}(i) = \mathbf{A}(t)\mathbf{k}(i)$

$$\begin{bmatrix} x(i,t_1) \\ x(i,t_2) \\ \vdots \\ x(i,t_L) \end{bmatrix} = \begin{bmatrix} a_f(t_1) & a_s(t_1) & a_p(t_1) \\ a_f(t_2) & a_s(t_2) & a_p(t_2) \\ \vdots & \vdots & \vdots \\ a_f(t_L) & a_s(t_L) & a_p(t_L) \end{bmatrix} \begin{bmatrix} k_f(i) \\ k_s(i) \\ k_p(i) \end{bmatrix}, \quad (2)$$

where the TACs associated with different APDs are

$$\begin{aligned} a_f(t) &= c_p(t) \otimes e^{-k_{2f}t}, \\ a_s(t) &= c_p(t) \otimes e^{-k_{2s}t}, \\ a_p(t) &= c_p(t), \end{aligned} \quad (3)$$

and $\otimes$ denotes the convolution operation. Relationship (2) describes how the observed multivariate data are generated by a process of mixing the latent components, as illustrated in Figure 2.

Since the APDs $\mathbf{k}(i)$ and TACs $\mathbf{A}(t)$ are both unknown, what we seek in the above model is an algorithm that can perform blind source separation to recover the source patterns from their observed mixtures. Based on the realistic assumption that the APDs are spatially heterogeneous (e.g., piecewise stationary with insignificant spatial overlap) [2, 3, 16], CCA on $\mathbf{x}(i)$ over the time domain aims to perform a nonparametric multivariate clustering of pixel TACs similarly to the successful application in functional MRI analysis [13]. Intuitively, when there are only pure-volume pixels, a one-to-one association between pixel TAC $x(i,t)$ and one of the source TACs $a_j(t)$ exists—except for a local scaling by $k_j(i)$ and some additive statistical variation

$$\mathbf{x}(i) = k_j(i)\mathbf{a}_j + \boldsymbol{\varepsilon}(i), \quad j \in \{f, s, p\}, \quad (4)$$

where $\boldsymbol{\varepsilon}(i)$ is the noise term being both temporally and spatially white Gaussian distributed with zero mean and unknown variance $\sigma^2_{x(i)}$ and $\mathbf{a}_j = [a_j(t_1), a_j(t_2), \ldots, a_j(t_L)]^T$ [13]. However, note that the forms of compartment TACs in (4) are no longer necessarily parametric as in (3) and are much more flexible; this should help reduce the potential for modeling failures. To perform a top-down CCA on $\mathbf{x}(i)$ to estimate $\mathbf{a}_j$ based on (4), the *shape* rather than the magnitude $k_j(i)$ of the pixel TAC is of the interest [7, 11, 17]. By performing both "centering" and "normalization" over time, given by

$$x_n(i,t) = \frac{1}{\sigma_{x(i)}}\left[x(i,t) - \frac{1}{L}\sum_{l=1}^{L} x(i,t_l)\right], \quad (5)$$

each pixel TAC can be transformed to a constant scale with mean zero independent of amplitude variations, denoted by the normalized $\mathbf{x}_n(i)$. There has been considerable success in using the standard finite normal mixture (SFNM) distribution to model clustered data sets, taking a sum of the following general form [18]:

$$\begin{aligned} p(\mathbf{x}_n(i)) &= \sum_{j}^{f,s,p} \pi_j g(\mathbf{x}_n(i) \mid \mathbf{a}_j, \mathbf{C}_j) \\ &= \sum_{j}^{f,s,p} \frac{\pi_j}{(2\pi)^{L/2}\left|\mathbf{C}_j\right|^{1/2}} \\ &\quad \times \exp\left(-\frac{1}{2}(\mathbf{x}_n(i) - \mathbf{a}_j)^T \mathbf{C}_j^{-1}(\mathbf{x}_n(i) - \mathbf{a}_j)\right), \end{aligned} \quad (6)$$

where $\pi$ is the mixing factor and $g$ is the Gaussian kernel with mean $\mathbf{a}_j$ and covariance matrix $\mathbf{C}_j$.

Finding an estimate of the mixing matrix $\mathbf{A}$ comes down to performing a maximum likelihood estimation of the SFNM model (6), where the joint log-likelihood is given by

$$\begin{aligned} \Phi(\mathbf{X}_n) = \sum_{i=1}^{N} \log \Bigg( \sum_{j}^{f,s,p} & \frac{\pi_j}{(2\pi)^{L/2}\left|\mathbf{C}_j\right|^{1/2}} \\ & \times \exp\left(-\frac{1}{2}(\mathbf{x}_n(i) - \mathbf{a}_j)^T \mathbf{C}_j^{-1}(\mathbf{x}_n(i) - \mathbf{a}_j)\right)\Bigg), \end{aligned} \quad (7)$$

where $N$ is the number of the pixels. This clustered component analysis task can be, fortunately, solved by the expectation-maximization (EM) algorithm that maximizes the joint log-likelihood [13, 15, 18]

$$\{\pi_j, \mathbf{a}_j, \mathbf{C}_j\} = \arg\max_{\pi_j, \mathbf{a}_j, \mathbf{C}_j} \Phi(\mathbf{X}_n \mid \pi_j, \mathbf{a}_j, \mathbf{C}_j), \quad (8)$$

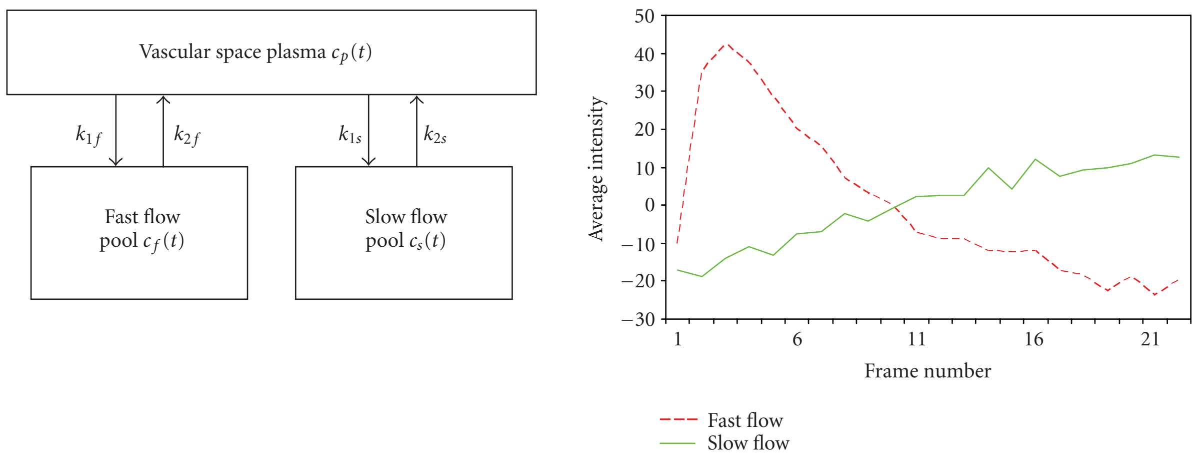

FIGURE 1: Schematic diagram of two-tissue compartment model and time-activity curves of fast and slow diffusions for quantifying tumor vascular characteristics based on DCE-MRI. The patterns of interest include the heterogeneous spatial distribution of vascular permeability associated with fast and slow diffusions.

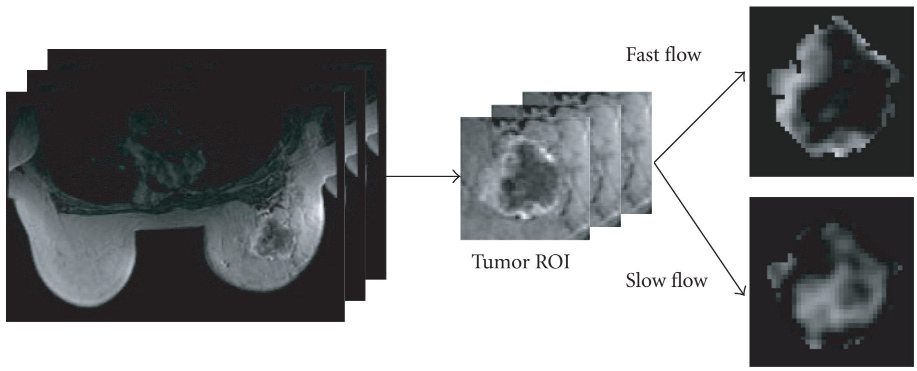

FIGURE 2: Illustration of source pattern mixing process.

where the "soft" splits of a pixel TAC allow $\mathbf{x}_n(i)$ to contribute simultaneously to multiple source TACs. Specifically, in order to compute the expectation step of the EM algorithm, we must first estimate the posterior probability that each pixel TAC $\mathbf{x}_n(i)$ is of source TAC $\mathbf{a}_j$, namely, the maximization step of the EM algorithm [13]. Such estimated posterior Bayes probabilities of pixel TAC $\mathbf{x}_n(i)$ associated with one of the source TACs are given by

$$z_{ij} = P(j \mid \mathbf{x}_n(i)) = \frac{\pi_j g(\mathbf{x}_n(i) \mid \mathbf{a}_j, \mathbf{C}_j)}{p(\mathbf{x}_n(i))}, \quad j \in \{f, s, p\} \quad (9)$$

and the compartment TACs are the normalized and weighted sample averages of pixel TACs in the light of their compartment memberships estimated by (9), computed via the expectation step of the EM algorithm [13]:

$$\mathbf{a}_j = \frac{\sum_{i=1}^{N} z_{ij}\mathbf{x}_n(i)}{\sum_{i=1}^{N} z_{ij}} \quad (10)$$

for $j = f, s, p$. Having determined the mixing matrix $\mathbf{A} = [\mathbf{a}_f, \mathbf{a}_s, \mathbf{a}_p]$ representing the compartment TACs, the APDs can be reconstructed using a least squares fit according to (2), resulting in

$$\hat{\mathbf{k}}(i) = (\mathbf{A}^T\mathbf{A})^{-1}\mathbf{A}^T\mathbf{x}(i), \quad (11)$$

where $T$ denotes matrix transpose.

To perform CCA, we use the visual statistical data analyzer (VISDA) algorithm [18]. The main function of VISDA is cluster modeling, discovery, and visualization. In addition to the multivariate soft clustering by the EM algorithm, VISDA also includes model selection by minimum description length (MDL) criterion and cluster initialization by hierarchical clustering. To capture all of the hidden clusters, VISDA is both statistically principled and visually insightful that incorporates both the power of statistical methods and the human gift for pattern recognition. VISDA uses an adaptive boosting of discriminatory subspaces involving hierarchical mixture modeling, selected optimally by the MDL criterion, and allows the complete data set to be visualized at the top level and so partitions data set, with clusters and subclusters of data points visualized at deeper levels.

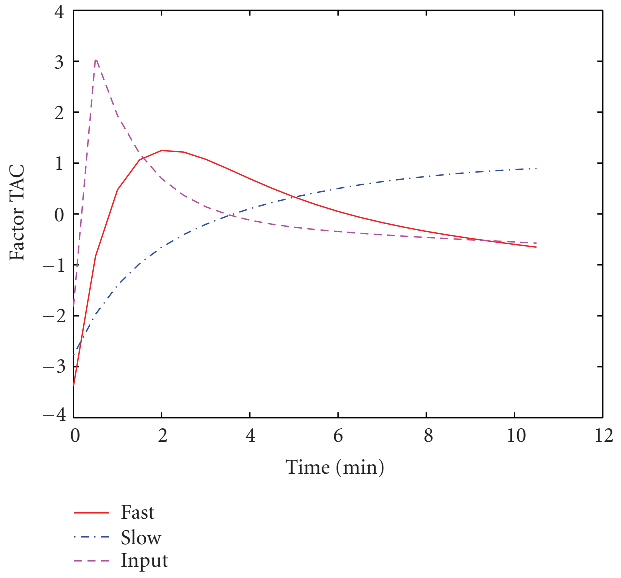

FIGURE 3: The estimated TACs derived from real DCE-MRI data by maximum likelihood method.

Complementary to (9) and (10), the M step in EM algorithm also involves the update rules for cluster factors and covariance matrices

$$\pi_j = \frac{1}{N}\sum_{i=1}^{N} z_{ij}, \qquad \mathbf{C}_j = \frac{\sum_{i=1}^{N} z_{ij}\left(\mathbf{x}_n(i)-\mathbf{a}_j\right)\left(\mathbf{x}_n(i)-\mathbf{a}_j\right)^T}{\sum_{i=1}^{N} z_{ij}} \tag{12}$$

for $j = f, s, p$. The E step involves assigning to the clusters, probabilistically, contributions from the data points and the M step involves re-estimating the parameters of the clusters in the light of this assignment. The algorithm cycles back and forth until the joint likelihood function is maximized.

When there are multiple compartment mixture regions, one remaining issue in pixel TAC clustering is the model selection that refers to the detection of cluster number $K_0$. The EM model fitting cannot be used to estimate $K_0$ since the ML is a nondecreasing function of $K_0$, thereby making it useless as a model selection criterion. This problem can be, fortunately, solved by using MDL criterion in conjunction with EM clustering. MDL is a proven information-theoretic criterion for model selection and has proven asymptotically consistent. The major thrust of MDL-based cluster validation has been the formulation of a model fitting procedure in which an optimal model is selected from the several competing candidates such that the selected model best fits the observed data. Specifically, the optimal value of $K_0$ is selected by minimizing

$$\mathrm{MDL}(K_0) = -\sum_{i=1}^{N}\log\left(\sum_{k=1}^{K_0}\pi_k g\left(\mathbf{x}_n(i) \mid \boldsymbol{a}_k, \mathbf{C}_k\right)\right)_{\mathrm{ML}} + \frac{6K_0-1}{2}\log N, \tag{13}$$

where the first term on the right is the approximation error and the second term on the right is the estimation error whose role is to penalize large value of $K_0$. For $K_0 = K_{\min}, \ldots, K_{\max}$, the values of MDL are calculated and a model with $K_0$ clusters is selected that will correspond to the minimum MDL value.

### 3. EXPERIMENT AND RESULTS

In this section, we first demonstrate the performance of clustered component analysis when applied to real DCE-MRI data sets. The data was acquired at the NIH Clinical Center using gadolinium DTPA as the contrast agent. The three-dimensional DCE-MRI scans were performed every 30 seconds for a total of 11 minutes after the injection. For the purpose of comparison, Figure 3 shows the estimated TACs associated with the input function as well as the fast and slow flows obtained by the advanced parametric compartment modeling method. The corresponding reconstructed APDs are given in Figure 4. This represents an advanced breast tumor case where active angiogenesis occurs often in the peripheral area (i.e., boundary with fast flow), while the inner core reflects hypoxia (dominated by slow flow) [2, 3].

We then apply CCA to the same data set. The DCE-MRI sequence contains a total of 18 images taken at different times, of which, we remove the first few images that do not show sufficient contrast accumulation and use the remaining 12–15 images in the experiment. After an analysis by VISDA, MDL criterion determines that there is clearly more than one pixel TAC cluster. By targeting the two major compartment sources, the corresponding source TACs are estimated and the hidden APD images are subsequently reconstructed. Figure 5 shows the extracted source images (i.e., vascular permeability) carrying out fast and slow diffusions. The projected distribution of pixel TACs clearly re-

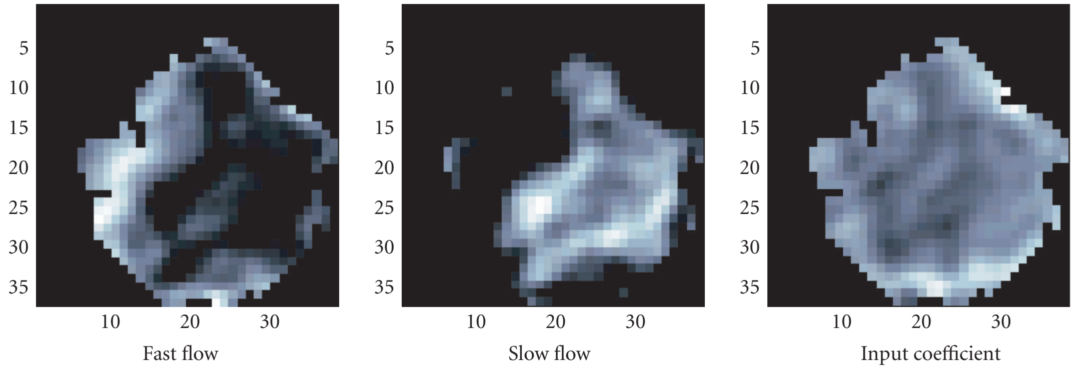

(a) (b) (c)

Figure 4: The reconstructed source factor images associated with fast and slow diffusions as well as plasma input.

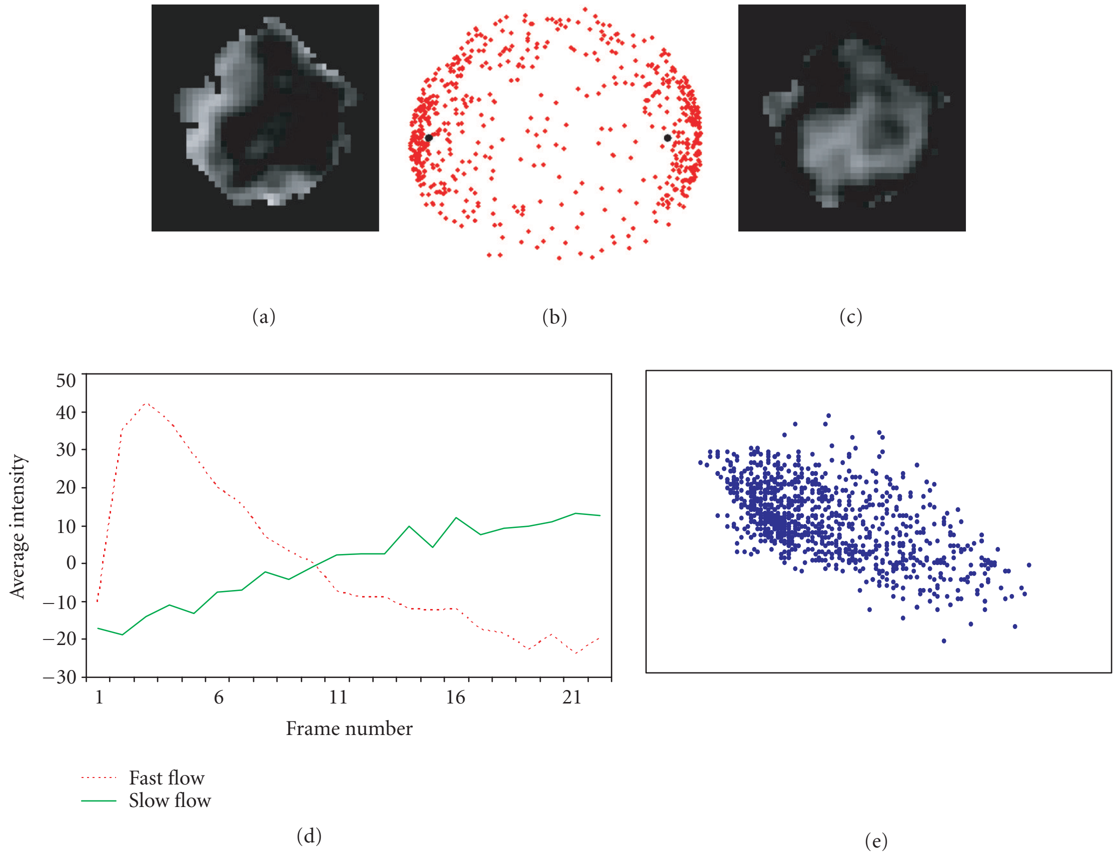

Figure 5: (b) shows the projected distribution of pixel TAC vectors (the dot plot represented in (b) is the two-dimensional projection of the component TAC clusters) whose two centers correspond to the fast and slow source TACs, respectively (the associated/extracted source images are given in (a) and (c)). (d) shows the kinetics of source TACs displayed as the time-course patterns and (e) shows the scatter plot of the source images showing the correlation patterns.

veals a multicluster data structure, and the scatter plot of the source images shows the expected globally negative correlation dependence.In addition, the ability of estimating the input function has aprofound impact in multivariate quantifications, since otherwise blood samples will be taken invasively at the radial artery or from an arterialized vein which, however, poses health risks and is not compatible with clinical practice. The outcome of CCA on further decomposing

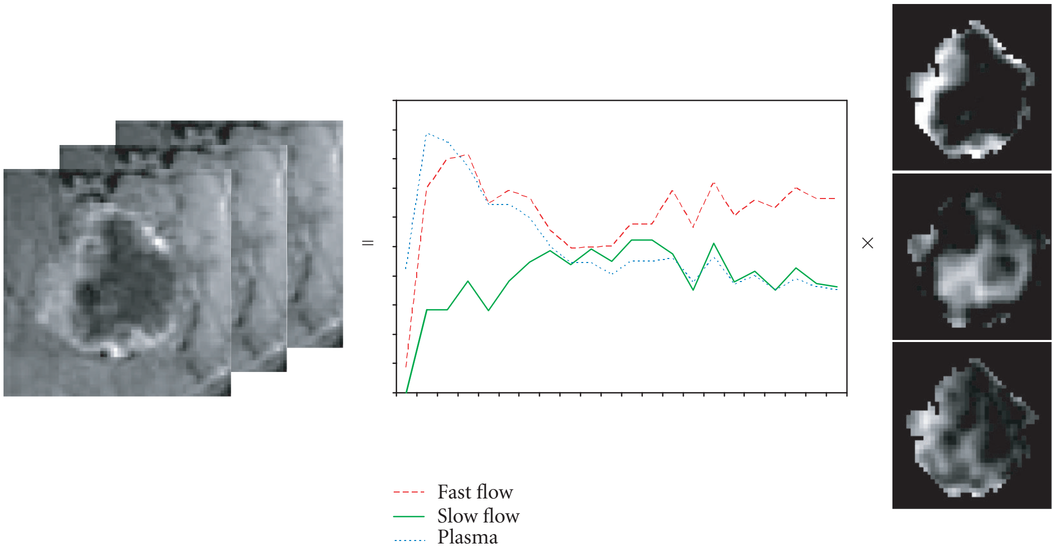

Figure 6: Compartmental latent variable modeling by CCA including plasma input. The source images are given in the right column (from top to bottom: fast flow, slow flow, and plasma input).

the mixture into the three underlying compartments is given in Figure 6 that presents a very consistent result with the one obtained by the independent method based on the compartment modeling shown in Figures 3 and 4. To test the stability of the performance by CCA, we have applied this method to a series of realistically simulated data sets in which various realistic TACs are numerically synthesized and the mixed observations are generated by weighting the real APDs (e.g., given in Figures 4 and 6) by these synthesized TACs. The results show that CCA can successfully reconstruct the hidden clustered components under various mixtures and TAC conditions.

As an example of more challenging problems, with significant practical utility, we report the preliminary application of CCA method in a longitudinal study to monitor a breast tumor's response to anti-angiogenic therapy. Defective endothelial barrier function due to vascular endothelial growth factor (VEGF) expression is one of the best-documented abnormalities of tumor vessels, resulting in spatially heterogeneous high microvascular permeability to macromolecules. Initial results suggest that changes in vascular permeability and volume fraction can be detected in a responsive tumor soon after therapy begins. Vascular permeability has been reported to correlate closely with VEGF expression in tumors, and decrease significantly after anti-VEGF antibody treatment and after the administration of other inhibitors of VEGF signaling. In breast and cervical cancers, a decrease in transendothelial permeability often accompanies tumor's response to chemotherapy and an early increase or no change in permeability can predict non-responsiveness or poorer prognosis.

Three sets of DCE-MRI data were acquired before and during the treatment period, each with three-months apart. Figure 7 shows the DCE-MRI images as a potential endpoint in assessing the response to therapy. The introduction of imaging cancer therapies by DCE-MRI has posed new challenges to traditional anatomic imaging approaches, because the vascularity of a tumor can change without a corresponding change in tumor size and vice versa. Our preliminary experiment shows promising results on the application of CCA to this problem, see Figure 8. For example, the extracted source TACs closely resemble the expected compartmental kinetics of the contrast agent, and both the APD images and TACs show the expected changes of the patterns over time, consistent with clinical assessment of a responsive case. Of particular scientific value, our results show that tumor-induced vascular activities were significantly reduced after a positive response to anti-angiogenesis chemotherapy, despite a noticeable increase in tumor volume during the initial treatment period.

### 4. DISCUSSION AND FUTURE WORK

In CCA approach, the significant overlap between the source image boundaries in space causes a potential partial volume effect (PVE) [16]. It can be shown that PVE will lead to a biased estimation of the compartment TACs. We can incorporate such PVE into the SFNM model that can be, fortunately, estimated by a constrained EM algorithm [16]. Specifically, we will first apply MDL criterion (13) to estimate the most appropriate number of distinctive temporal clusters that will also include the so-called composite boundary clusters [16]; we will then estimate the PVE-SFNM model by only updating the parameters of pure-volume clusters using$z_{ij}$, followed by the assignment of the parameter values for the partial volume clusters based on the PVE model [16]. Alternatively, we can simply consider those composite boundary clusters as intrinsic compartment TACs where the emphasis will be on

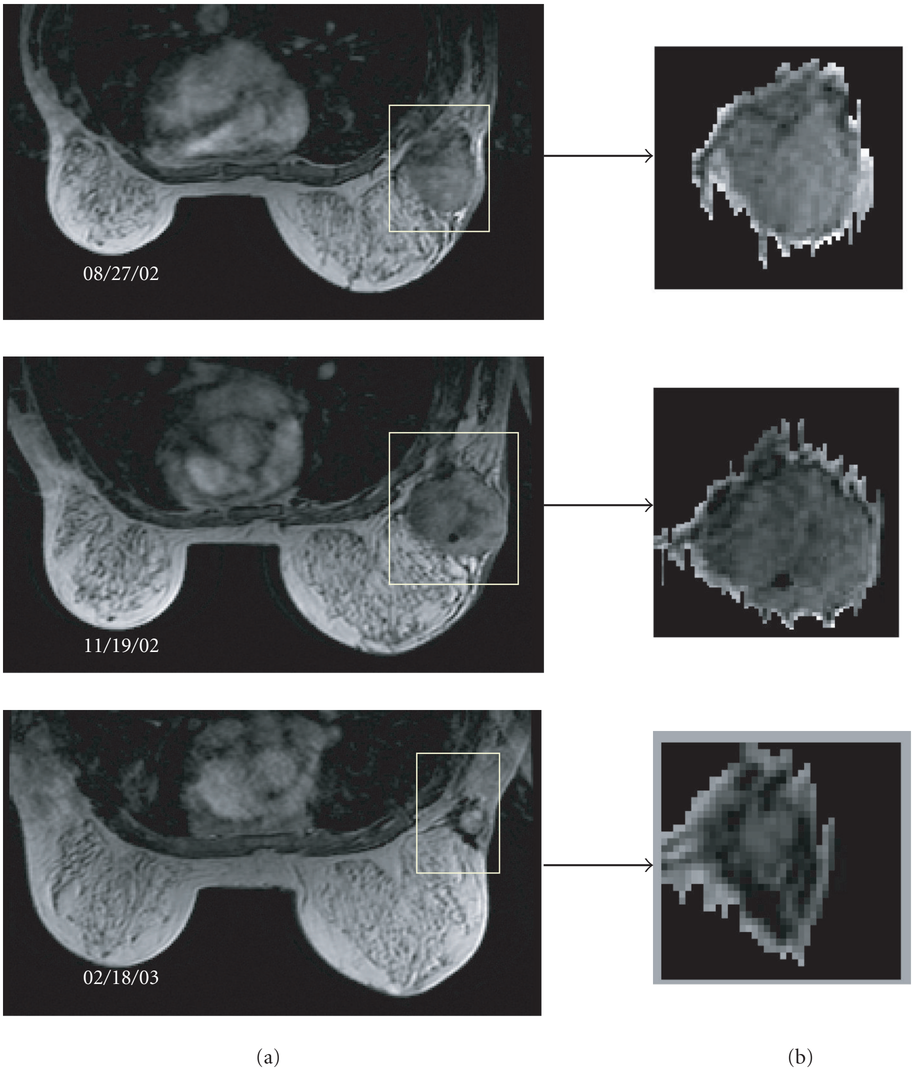

Figure 7: DCE-MRI as a potential endpoint in monitoring tumor's response to anti-angiogenic therapy. Three sets of DCE-MRI data of the same tumor were acquired and are shown in (a). The tumor sites were extracted via advanced image segmentation tools and are highlighted in (b). The pattern changes are consistent with the clinical foundings that, even as a responsive case, most tumors' volume will grow initially but shrink lately with much reduced vascular activities.

interpreting such compartments in relation to biological or clinical parameters.

In longitudinal studies, special care should be taken in assessing the response to therapy. Inour present experiment, we have considered longitudinal samplings separately and performed blind source separation for each of the samplings. It would be more meaningful to consider the estimated TAC before the therapy starts as a baseline reference, and then estimate the source images in the follow-up studies to see whether the spatial distribution of fast and slow permeabilities changes. We can also use the estimated baseline source image as the reference, and subsequently recover the TACs in the follow-up studies to detect the changes of diffusion rates. The multivariate quantifications that reflect the efficacy of angiogenesis inhibitors has great potential but is at an early stage. Part of the challenge stems from incomplete knowledge of how blood vessels are affected. For example, angiogenesis inhibitors can block the growth of new blood vessels from existing vessels, but may also eliminate certain existing vessels, such as tumor vessels.

We believe that our comparative studies provide useful information on the utility of the proposed methods for computed simultaneous imaging of multiple functional or molecular biomarkers. Given the difficulty of the task, while the optimality of these methods may be data or modality dependent, we wouldexpect them to be important toolsin dynamic image formation and analysis. For example, since angiogenesis is complex process critical to growth and metastasis of malignant tumors, the clinical value and promise of DCE-MRI in imaging tumor angiogenesis before

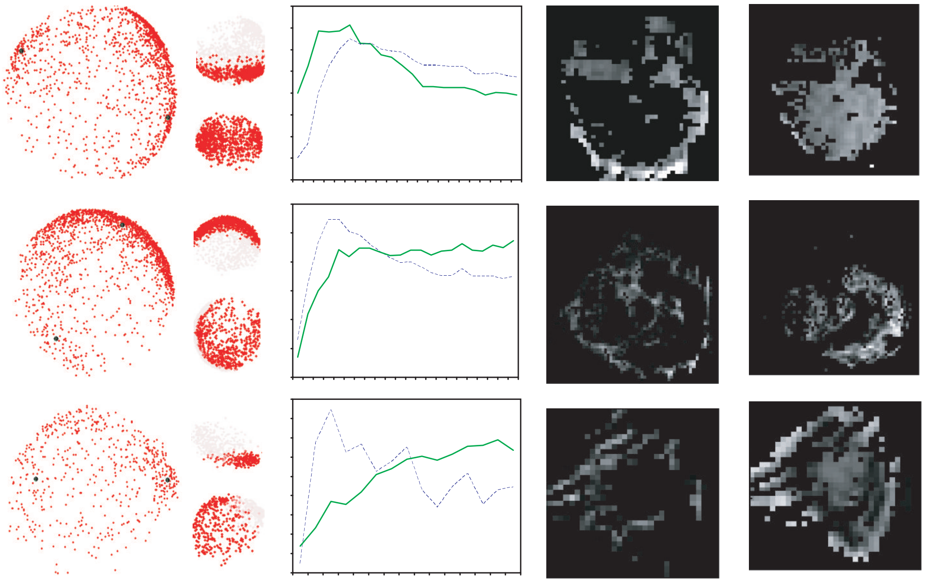

Figure 8: Blind decomposition of permeability distribution and diffusion dynamics via CCA in a longitudinal study with three-time snapshots shown in Figure 7. (a) 2D projection of compartment TAC clusters and 2D projection of individual compartment TAC clusters; (b) TACs corresponding to fast and slow flows/perfusions; and (c) and (d) extracted angiogenic permeability distributions (source images) associated with fast and slow flows/perfusions. Serving as the quantitative measures for monitoring functional response to therapy, the results correspond to a positive responsive case where both fast and slow diffusion/perfusion rates are significantly reduced during and after the therapy.

and during therapy provides strong incentive for advancing the imaging formation method [2–4]. Specifically, with the prior information on the nonnegativity of the mixing matrix and sources, new principle and perhaps improved methods may yet become possible [19]. Here we wish to propose a nonnegative least-correlated component analysis (nLCA) when the hidden sources and mixing matrix are known to be nonnegative [20]. This concept has powerful features which are of considerable universal applicability since it eliminates the condition of source independence and non-Gaussianity required by independent component analysis [19]. It can be shown that when the mixing matrix is nonnegative, the correlation between the mixtures is always positively increased, namely, the *correlation increase theorem* [20]. Such a positive increase in correlation after nonnegative mixing immediately suggests a possible recovering mechanism for blind source separation of dependent sources. With the encouraging preliminary success tested on real data sets, we are currently investigating the existence and uniqueness of nLCA solution that exploits the nonnegativity constraint on correlated yet well-grounded sources in the light of correlation increase theorem [19, 20].

### ACKNOWLEDGMENT

This work was supported in part by the National Institutes of Health under Grant EB000830 and the Department of Defense under Grant DAMD17-98-8045.

### REFERENCES

[1] H. R. Herschman, “Molecular imaging: looking at problems, seeing solutions,” *Science*, vol. 302, no. 5645, pp. 605–608, 2003.

[2] D. M. McDonald and P. L. Choyke, “Imaging of angiogenesis: from microscope to clinic,” *Nature Medicine*, vol. 9, no. 6, pp. 713–725, 2003.

[3] A. R. Padhani and J. E. Husband, “Dynamic contrast-enhanced MRI studies in oncology with an emphasis on quantification, validation and human studies,” *Clinical Radiology*, vol. 56, no. 8, pp. 607–620, 2001.

[4] N. Hylton, “Magnetic resonance imaging of the breast: opportunities to improve breast cancer management,” *Journal of Clinical Oncology*, vol. 23, no. 8, pp. 1678–1684, 2005.

[5] G. J. Parker, J. Suckling, S. F. Tanner, et al., “Probing tumor microvascularity by measurement, analysis and display of contrast agent uptake kinetics,” *Journal of Magnetic Resonance Imaging*, vol. 7, no. 3, pp. 564–574, 1997.

[6] D. Y. Riabkov and E. V. R. Di Bella, “Estimation of kinetic parameters without input functions: analysis of three methods for multichannel blind identification,” *IEEE Transactions on Biomedical Engineering*, vol. 49, no. 11, pp. 1318–1327, 2002.

[7] Y. Zhou, S. C. Huang, T. Cloughesy, C. K. Hoh, K. Black, and M. E. Phelps, “A modeling-based factor extraction method for determining spatial heterogeneity of Ga-68 EDTA kinetics in brain tumors,” *IEEE Transactions on Nuclear Science*, vol. 44, no. 6, part 2, pp. 2522–2526, 1997.

[8] K.-P. Wong, S. R. Meikle, D. Feng, and M. J. Fulham, “Estimation of input function and kinetic parameters using simulated annealing: application in a flow model," *IEEE Transactions on Nuclear Science*, vol. 49, no. 3, part 1, pp. 707–713, 2002.

[9] Z. J. Wang, Z. Han, K. J. R. Liu, and Y. Wang, "Simultaneous estimation of kinetic parameters and the input function from DCE-MRI data: theory and simulation," in *Proceedings of IEEE International Symposium on Biomedical Imaging: Macro to Nano (ISBI '04)*, vol. 1, pp. 996–999, Arlington, Va, USA, April 2004.

[10] G. Pillonetto, G. Sparacino, and C. Cobelli, "Numerical non-identifiability regions of the minimal model of glucose kinetics: superiority of Bayesian estimation," *Mathematical Biosciences*, vol. 184, no. 1, pp. 53–67, 2003.

[11] N. Keshava and J. F. Mustard, "Spectral unmixing," *IEEE Signal Processing Magazine*, vol. 19, no. 1, pp. 44–57, 2002.

[12] B. S. Everitt, *An Introduction to Latent Variable Models*, Chapman & Hall, London, UK, 1984.

[13] S. Chen, C. A. Bouman, and M. J. Lowe, "Clustered components analysis for functional MRI," *IEEE Transactions on Medical Imaging*, vol. 23, no. 1, pp. 85–98, 2004.

[14] Y. Wang, J. Zhang, K. Huang, J. Khan, and Z. Szabo, "Independent component imaging of disease signatures," in *Proceedings of IEEE International Symposium on Biomedical Imaging (ISBI '02)*, pp. 457–460, Washington, DC, USA, July 2002.

[15] Y. Wang, R. Srikanchana, P. L. Choyke, J. Xuan, and Z. Szabo, "Computed simultaneous imaging of multiple functional biomarkers," in *Proceedings of IEEE International Symposium on Biomedical Imaging: Macro to Nano (ISBI '04)*, vol. 1, pp. 225–228, Arlington, Va, USA, April 2004.

[16] P. Santago and H. D. Gage, "Statistical models of partial volume effect," *IEEE Transactions on Image Processing*, vol. 4, no. 11, pp. 1531–1540, 1995.

[17] P. Tamayo, D. Slonim, J. Mesirov, et al., "Interpreting patterns of gene expression with self-organizing maps: methods and application to hematopoietic differentiation," *Proceedings of the National Academy of Sciences of the United States of America*, vol. 96, no. 6, pp. 2907–2912, 1999.

[18] Y. Wang, L. Luo, M. T. Freedman, and S.-Y. Kung, "Probabilistic principal component subspaces: a hierarchical finite mixture model for data visualization," *IEEE Transactions on Neural Networks*, vol. 11, no. 3, pp. 625–636, 2000.

[19] E. Oja and M. Plumbley, "Blind separation of positive sources by globally convergent gradient search," *Neural Computation*, vol. 16, no. 9, pp. 1811–1825, 2004.

[20] Y. Wang, "Nonnegative reverse correlation as guiding principle for universal blind source separation," in *Advanced Research Institute Technical Report*, S. Rahman, Ed., pp. 1–2, Virginia Tech, Arlington, Va, USA, 2003.

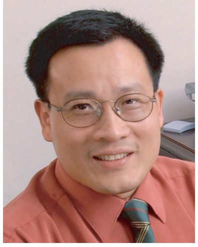

**Yue Wang** received his B.S. and M.S. degrees in electrical and computer engineering from Shanghai Jiao Tong University in 1984 and 1987, respectively. He received his Ph.D. degree in electrical engineering from University of Maryland Graduate School in 1995. In 1996, he was a Postdoctoral Fellow at, Georgetown University School of Medicine. From 1996 to 2003, he was an Assistant and later Associate Professor of electrical engineering, The Catholic University of America, Washington DC. Since 2003, he has been an Associate Professor of electrical, computer, and biomedical engineering at Virginia Polytechnic Institute and State University, Arlington, VA. He is also an affiliated faculty member of radiology and radiological science with the Johns Hopkins University. Yue Wang became a Fellow of The American Institute for Medical and Biological Engineering (AIMBE) in 2004. His research interests focus on intelligent computing, machine learning, pattern recognition, statistical visualization, and advanced imaging and image analysis, with applications to molecular analysis of human diseases.

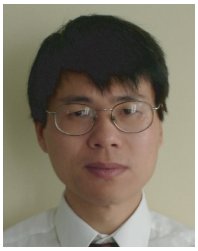

**Jianhua Xuan** received his Ph.D. degree in electrical engineering and computer science from the University of Maryland in 1997. He received his B.S., M.S., and Ph.D. degrees from University of Zhejiang, China, in 1985, 1988, and 1991, respectively, all in electrical engineering. Currently, he is an Assistant Professor in the Department of Electrical Engineering and Computer Science at The Catholic University of America. His research interests include biomedical image analysis, cellular and molecular imaging, computational bioinformatics, systems biology, intelligent information systems, visual intelligence, compute vision, information visualization, and machine learning. He is a recipient of several NIH/DoD/NASA grant awards, and leading a group's efforts in cancer research and biomedical research. He is also a Member of the editorial board for BioMedical Engineering OnLine.

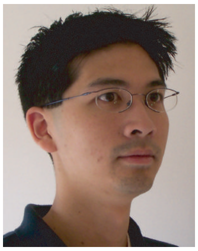

**Rujirutana Srikanchana** received his Ph.D. degree in electrical engineering from The Catholic University of America in 2003. Currently he is a Research Scientist at Riverain Medical, where he is engaged in research and development of a computer-aided detection (CAD) system to assist radiologists in detecting early-stage lung cancer on chest radiograph. His research interests include medical image analysis, machine learning, neural networks, and pattern recognition.

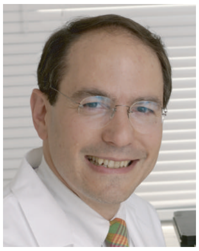

**Peter L. Choyke** received his M.D. degree from Jefferson Medical College. He completed his residency in Diagnostic Radiology at Yale-New Haven Hospital and his Fellowship in Abdominal Imaging at the University of Pennsylvania. He joined the faculty of Georgetown University Medical Center, where he was appointed an Assistant and then an Associate Professor of radiology. In 1988 he joined the Diagnostic Radiology Department in the Warren G. Magnuson Clinical Center of the National Institutes of Health in Bethesda, Maryland, where he specialized in genitourinary oncologic imaging. He became a Professor of radiology at the Uniformed Services University of the Health Sciences in 1990. In 2004 he was named the Chief of the Molecular Imaging Program within the National Cancer Institute where he is a Senior Clinician. His research interests focus on developing new clinical imaging methods and testing new molecular imaging probes for the diagnosis and monitoring of cancer and clinical imaging of urologic cancers.

